# Vitamin B1 Deficiency Identified from Incidental Detection of Hyperlactatemia: A Case Report

**DOI:** 10.3390/medicina60050715

**Published:** 2024-04-26

**Authors:** Yuki Omura, Koshi Ota, Akira Takasu, Tomio Suzuki

**Affiliations:** 1 Department of General Medicine, Osaka Medical and Pharmaceutical University, 2-7 Daigaku-machi, Takatsuki City 596-8686, Osaka, Japan; yuki.omura@ompu.ac.jp (Y.O.); tomio.suzuki@ompu.ac.jp (T.S.); 2 Department of Emergency and Critical Care Medicine, Osaka Medical and Pharmaceutical University, 2-7 Daigaku-machi, Takatsuki City 596-8686, Osaka, Japan; akira.takasu@ompu.ac.jp

**Keywords:** hyperlactatemia, vertigo, vitamin B1 deficiency, Wernicke’s encephalopathy

## Abstract

*Introduction*: Vitamin B1 deficiency poses a significant risk of impaired consciousness, with manifestations ranging from anorexia and fatigue to severe neurological and cardiovascular disturbances. Wernicke’s encephalopathy, a neurological disorder stemming from vitamin B1 deficiency, presents as the triad of ophthalmoplegia, altered mental state, and cerebellar ataxia. However, these symptoms are not consistently present, complicating the diagnosis. In addition, subclinical vitamin B1 deficiency can progress unnoticed until severe complications arise. Studies indicate a high rate of undiagnosed cases, emphasizing the need for early detection and intervention. *Case presentation*: We present the case of a 65-year-old man in whom hyperlactatemia was incidentally detected, leading to the diagnosis of vitamin B1 deficiency. The patient, presenting with vertigo and vomiting, had been eating boxed lunches bought from convenience stores following the death of his wife 3 years earlier. Vertigo gradually improved with rest, but the persistence of hyperlactatemia prompted further investigation, revealing low vitamin B1 levels and high pyruvate levels. Treatment with dietary adjustments and supplements significantly improved his symptoms. *Discussion*: In this case, hyperlactatemia was found in a vertigo patient, revealing asymptomatic vitamin B1 deficiency. Elevated lactate is often linked with conditions like sepsis but can also stem from overlooked factors such as low vitamin B1 levels due to poor diet habits like consuming fried foods. *Conclusion*: This case highlights the importance of considering vitamin B1 deficiency in patients with unexplained hyperlactatemia, even in high-income countries. Early detection can prevent progression to the severe complications associated with Wernicke’s encephalopathy. Proactive measurement of lactate levels in at-risk populations may facilitate early diagnosis and intervention, ultimately improving patient outcomes.

## 1. Introduction

Vitamin B1 deficiency should be considered as a potential cause of impaired consciousness. Manifestations of vitamin B1 deficiency include symptoms like anorexia, fatigue, and neurological and cardiovascular disturbances [1]. Left untreated, this deficiency can progress to severe encephalopathy.

Wernicke’s encephalopathy is a neurological disturbance caused by vitamin B1 deficiency. A typical case presents with the triad of ophthalmoplegia, altered mental state, and cerebellar ataxia. However, these symptoms are not disease-specific, with only 16% of patients developing all three signs and 19% remaining asymptomatic [2,3]. Subclinical vitamin B1 deficiency has also been reported, with vitamin B1 deficiency progressing in the absence of noticeable symptoms, only being detected after the development of Wernicke’s encephalopathy [3]. When all symptoms of vitamin B1 deficiency or Wernicke’s encephalopathy are absent, diagnosing vitamin B1 deficiency or Wernicke’s encephalopathy becomes much more challenging.

Alarmingly, studies have shown that as much as 80% of cases of Wernicke’s encephalopathy resulting from vitamin B1 deficiency may pass undiagnosed during the initial presentation, emphasizing the importance of early intervention and preventive measures before encephalopathy advances [4,5].

Vitamin B1 is a coenzyme that converts pyruvate to acetyl CoA in the glycolytic system. Pyruvate therefore accumulates when vitamin B1 is deficient, leading to the conversion of accumulated pyruvate into lactic acid.

Here, we present a case in which incidental detection of hyperlactatemia led to the early detection of vitamin B1 deficiency and successful treatment.

### Case Presentation

An otherwise healthy 65-year-old man presented to our emergency department complaining of vertigo and vomiting. One month previously, he had started experiencing episodes of vertigo that recurred 2 or 3 times during that period. However, he did not seek medical attention. Upon waking in the morning on the day of this visit, he experienced an episode of vertigo that lasted about 10 s but stopped when he lay down to rest. When he attempted to get up around noon, he again experienced vertigo, accompanied by vomiting, at which point he visited the emergency room.

The patient had no prior medical history or surgeries and denied smoking cigarettes, abusing alcohol, or using illicit drugs. Following the death of his wife 3 years earlier, he had only been eating deep-fried chicken in boxed lunches bought from convenience stores.

Physical examination revealed a middle-aged man of average build and clear mental status. Vital signs included temperature, 36.0 °C; heart rate, 70 beats/min; respiratory rate, 22 breaths/min; blood pressure, 126/86 mmHg; and oxygen saturation, 100% in ambient air. No rales were auscultated in either lung and heart sounds were normal. No abdominal tenderness or organomegaly was observed. Pupil diameters were normal, with no nystagmus or abnormalities of ocular position. No motor or sensory neuropathy was noted, and there was no evidence of hearing loss. Horizontal nystagmus accompanied by vertigo was induced when the patient was repositioned from supine to right lateral recumbency, with the vertigo lasting approximately 20 s.

Initial laboratory examination showed mild leukocytosis (white blood cell count, 9780/mm^3^; 69.9% neutrophils), but C-reactive protein levels were normal (0.07 mg/dL; reference value, 0–0.3 mg/dL). The patient showed no anemia (hemoglobin, 15.2 g/dL), thrombocytopenia (platelet count, 335,000/μL), renal failure (creatinine, 0.65 mg/dL), or liver failure (serum aspartate aminotransferase, 27 IU/L; serum alanine aminotransferase, 36 IU/L). No electrolyte abnormalities or obvious diabetes were detected (sodium, 138 mmol/L; potassium, 3.6 mmol/L; chloride, 103 mmol/L; blood glucose level, 164 mg/dL).

Arterial blood gases revealed respiratory alkalosis due to an increased respiratory rate (pH 7.503; pCO2, 23 mmHg; pO2, 119 mmHg in ambient air; HCO3-, 17.7 mmol/L; base excess, −3.3 mmol/L) and metabolic acidosis due to a widening anion gap, with hyperlactatemia (anion gap, 19.6 mmol/L; lactate, 49.2 mg/dL). Computed tomography (CT) of the head ruled out cerebral hemorrhage, cerebral infarction, and space-occupying lesions. Echocardiography showed no abnormal findings. Initial laboratory examination results are shown in Table 1.

The patient was kept at rest in the emergency room and vertigo gradually improved. Despite administration of 500 mL of saline, hyperlactatemia persisted in arterial blood gases (lactate, 58.0 mg/dL). The patient remained in good general condition and was discharged from the emergency room without the need for admission to the hospital. The patient was sent to an outpatient department of internal medicine for follow-up and diagnosis. Arterial blood gases continued to show persistent hyperlactatemia the day after the emergency room visit (lactate, 52.4 mg/dL) as well as one week later (lactate, 32.6 mg/dL). Physical examination at that time revealed decreased patellar and Achilles tendon reflexes. Laboratory examination also indicated a low vitamin B1 level (22 ng/mL; reference value, 24–66 ng/mL) and a high pyruvate level (1.72 mg/dL; reference value, 0.30–0.94 mg/dL). Hyperlactatemia was attributed to vitamin B1 deficiency, so the patient was provided instructions on improving his diet and taking supplements.

**Figure 1:**
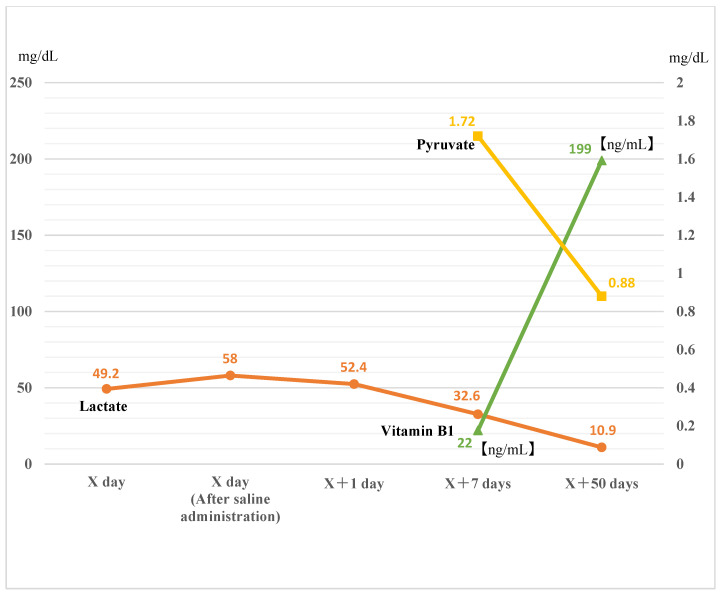
Lactate, vitamin B1, and pyruvate over time. At the time of the emergency room visit, lactate levels were high. Seven days after the visit, vitamin B1 levels were found to be low. Lactate and pyruvate levels tended to decrease after vitamin B1 supplementation. X = Date of first visit to the emergency room.

Laboratory examination performed 50 days after the initial emergency room visit showed improved levels of lactate (10.9 mg/dL), vitamin B1 (199 ng/mL), and pyruvate (0.88 mg/dL), along with the normalization of tendon reflexes. At this point, the follow-up was terminated. Changes in concentrations of lactate, vitamin B1, and pyruvate over the clinical course are shown in Figure 1.

## 2. Discussion

In this case, hyperlactatemia was incidentally discovered in a patient who presented with a chief complaint of vertigo, leading to the diagnosis of asymptomatic vitamin B1 deficiency.

Elevated lactate levels can have numerous causes. Hyperlactatemia is classified into Type A and Type B depending on the cause. Type A, the most severe form of the disease, results from the excessive production of lactic acid in ischemic tissues as a byproduct of anaerobic glycolysis to produce adenosine triphosphate during oxygen deprivation. Type B occurs with normal overall tissue perfusion, and the prognosis is not so bad. The causes of Type A and Type B are shown in Table 2. The majority of the medical literature on the significance of lactate levels has predominantly focused on septic shock, and this literature-based selection bias may predispose clinicians to associate elevated lactate solely with sepsis. However, any form of shock or tissue hypoperfusion can result in elevated lactate. Several other causes of elevated lactate also exist, independent of these states [6]. In particular, elevated lactate resulting from vitamin B1 deficiency is often overlooked. Vitamin B1 serves as a cofactor for multiple cellular enzymes, including α-ketoglutarate dehydrogenase and pyruvate dehydrogenase, which are essential components of both aerobic carbohydrate metabolism and the tricarboxylic acid cycle. Vitamin B1 facilitates the conversion of pyruvate to acetyl CoA in the glycolytic system. Anaerobic metabolism predominates in the absence of vitamin B1, leading to the accumulation of pyruvate and its subsequent conversion into lactic acid. The development of elevated lactate in serum secondary to vitamin B1 deficiency has been described in detail [1,6].

Vitamin B1 deficiency has typically been associated with alcoholism or as a prevalent problem in low- and middle-income countries with populations that rely on staple foods with a low content of thiamine [4]. This may explain why vitamin B1 deficiency is often overlooked in high-income countries like Japan. However, many reports have described vitamin B1 deficiency in high-income countries [4]. In such countries, vitamin B1 deficiency can arise in patients with heart failure, cancer, gastrointestinal diseases, inappropriately formulated parenteral nutrition, or surgeries involving the gastrointestinal tract that cause reduced absorption of nutrients [4].

Vitamin B1 deficiency can occur even in non-drinkers with inadequate nutrition [7]. Inadequate nutrition can result from picky eating, as in the present case, and picky eaters are more likely to be found in high-income countries with no shortage of food compared to low-income countries. For example, young people who live alone and are busy with work tend to eat the same kind of food, because these groups often buy easy-to-prepare or ready-made foods. Individuals on excessively strict diets also tend to eat less, which can lead to nutritional deficiencies [8]. The daily requirement for vitamin B1 for a 65-year-old man is 1.3 mg [9]. While 100 g of chicken is estimated to contain 0.1 mg of vitamin B1, chicken loses as much as 55% of its vitamin B1 content when fried [10]. The patient in this case, who had eaten only deep-fried chicken in boxed lunches from convenience stores for 3 years, had probably experienced mild but long-term vitamin B1 deficiency. People with lifestyle habits as seen in this case are thought to be more common in relatively high-income countries. Thus, in high-income countries, these lifestyle habits should be kept in mind as a cause of vitamin B1 deficiency.

The proactive measurement of lactate levels in patients with these risk factors may facilitate the early detection of vitamin B1 deficiency. Although vitamin B1 deficiency and Wernicke’s encephalopathy are generally associated with a variety of neurological symptoms, many asymptomatic cases have been reported. Vertigo is considered a late manifestation of vitamin B1 deficiency. We could not identify any case reports of early vitamin B1 deficiency causing vertigo alone, as in this case. The early detection of Wernicke’s encephalopathy before it progresses, even when asymptomatic, is crucial, and the measurement of lactate levels may be useful for this purpose.

## 3. Conclusions

In the present case, vitamin B1 deficiency was identified from incidental detection of hyperlactatemia and attributed to an unbalanced diet. Vitamin B1 deficiency can occur even in high-income countries if the diet is unbalanced. Vitamin B1 deficiency is easily overlooked and should be kept in mind whenever hyperlactatemia is identified.

## Figures and Tables

**Table 1:** Initial laboratory examination.

Laboratory Examination			Arterial Blood Gases		
TP	7.2	g/dL	pH	7.503	
Alb	3.9	g/dL	PaCO2	23	mmHg
AST	27	g/dL	PaO2	119	mmHg
ALT	36	U/L	HCO3-	17.7	mmol/L
LD	137	U/L	AnGap	19.6	mmol/L
ALP	101	U/L	Lactate	49.2	mg/dL
γ-GTP	15	U/L			
CK	27	U/L			
BUN	8	mg/dL			
Cre	0.65	mg/dL			
CRP	0.07	mg/dL			
Blood glucose	164	mg/dL			
Na	138	mmol/L			
K	3.6	mmol/L			
Cl	103	mmol/L			
WBC	9780	/μL			
Hb	15.2	g/dL			
Plt	335	(10)3/μL			

**Table 2:** The cause of TypeA and TypeB hyperlactemia.

TypeA	TypeB
Hypovolemic shock	Diabate
Cardiogenic shock	Medicinal (Metformin, Theophylline, Adrenaline, Methanol, Propofol, etc.)
Septic shock	Vitamin B1 deficiency
Anemia	Alcoholism
Hypoxemia	Malignancy
Trauma and Burns	Short bowel syndrome
Compartment syndrome	Cirrhosis
Non-occlusive mesenteric ischemia	Congenital disease
Shivering	
Spasm	

## Data Availability

The datasets used in the current study are available from the corresponding author on reasonable request.
